# What shapes participation in cerebral palsy? A Co-produced PEM-CY scoping review with mothers with lived experience

**DOI:** 10.1186/s12887-026-07282-8

**Published:** 2026-07-27

**Authors:** Jaíza Marques Medeiros e Silva, Jean Bendito Félix, Viviann Alves de Pontes, Lívia Ferreira Coutinho Alonso, Michele Gomes da Silva, Mayra Conceição Lima da Silva Paiva, Hércules Ribeiro Leite, Kennea Martins Almeida Ayupe, Rosane Luzia de Souza Morais, Paula Silva de Carvalho Chagas, Ana Cristina Resende Camargos, Ana Carolina de Campos, Rafaela Silva Moreira, Luiz Medeiros Araujo Lima-Filho, Egmar Longo

**Affiliations:** 1 Universidade Federal da Paraíba (UFPB), João Pessoa, Paraíba Brasil; 2 Universidade Federal de Juiz de Fora (UFJF), Juiz de Fora, Minas Gerais Brasil; 3 Family Partner, Juiz de Fora, Minas Gerais Brasil; 4 Family Partner, João Pessoa, Paraíba Brasil; 5 Universidade Federal de Minas Gerais (UFMG), Belo Horizonte, Minas Gerais Brasil; 6 Universidade Federal do Espírito Santo (UFES), Vitória, Espírito Santo Brasil; 7 Universidade Federal dos Vales do Jequitinhonha e Mucuri (UFVJM), Diamantina, Minas Gerais Brasil; 8 Universidade Federal de São Carlos (UFSCar), São Carlos, São Paulo, Brasil; 9 Universidade Federal de Santa Catarina (UFSC), Florianópolis, Santa Catarina Brasil

**Keywords:** Participation, Environment, Cerebral Palsy, Children, Adolescents, Patient and Public Involvement

## Abstract

**Background:**

Children and adolescents with cerebral palsy (CP) may experience restricted participation across different settings. Although several instruments assess participation, the *Participation and Environment Measure for Children and Youth* (PEM-CY), a caregiver-report instrument, is a robust tool for evaluating both Participation and Environment. This study aimed to map Participation and Environment in the home, school, and community settings of children and adolescents with CP using the PEM-CY, in partnership with a Public and Patient Involvement (PPI) group, to ensure that the review reflects the experiences and priorities of families.

**Methods:**

This scoping review was structured according to the guidelines provided by the Joanna Briggs Institute (JBI) and adapted to incorporate contributions from the PPI group, comprising mothers of children with CP, three physiotherapists, and one occupational therapist. Partner mothers contributed to developing the guiding research question, selecting search keywords, reviewing the results, interpreting the findings, and refining the review’s implications and conclusions. Searches were conducted in six electronic databases (Medline/PubMed, Embase, Scopus, Web of Science, Lilacs, and CINAHL). We included original observational studies that examined Participation and Environment using the PEM-CY. Data were collected using a custom extraction form and presented in tables and figures.

**Results:**

Twelve studies from different countries were included. Participation was higher at home and lower in the community, both in frequency and involvement, while the desire for change was most pronounced in community activities. Children with CP showed lower participation and less environmental support than peers without disabilities, and higher motor function and physical activity were associated with greater participation.

**Conclusion:**

Children and adolescents with CP face greater challenges in participating in activities, particularly in community settings, when compared to their peers without disabilities. Higher levels of physical activity, environmental adaptation, and family and clinical support increases participation among children and adolescents with CP. The inclusion of the PPI group added social relevance and sensitivity to the theoretical process of the review, helping ensure that the findings better reflect the needs and priorities of families. Retrospectively registered: 10.17605/OSF.IO/VPQM5.

**Supplementary Information:**

The online version contains supplementary material available at https://doi.org/10.1186/s12887-026-07282-8.

## Introduction

Cerebral palsy (CP) is considered an early-onset, permanent neurodevelopmental condition characterized by limitations in activities due to impaired motor and postural development [1]. These limitations result from a non-progressive brain lesion or malformation, although the manifestations of CP may change throughout development [1]. These activity limitations can negatively impact participation in different settings, such as home, school, and community [2].

Participation is defined by the International Classification of Functioning, Disability and Health (ICF) as the involvement of an individual in real-life situations. This framework also specifies domains of participation that can be assessed, including community, social, and civic life [3]. However, the family of Participation-Related Constructs (fPRC) was proposed to further clarify the constructs associated with participation, addressing the lack of specificity in the ICF definition, and distinguishes key components such as attendance and involvement in activities, as well as intrinsic (child-related) and extrinsic (contextual) factors that influence participation [4].

Currently, several instruments are available for evaluating participation in children and adolescents with CP, such as the Children’s Assessment of Participation and Enjoyment (CAPE) [5, 6], the Assessment of Life Habits (LIFE-H) [7], the Preferences for Activities of Children (PAC) [5], and the Children Helping Out: Responsibilities, Expectations, and Supports (CHORES) [8]. However, these tools do not assess participation across different environments such as home, school, and community. The Participation and Environment Measure for Children and Youth (PEM-CY) was specifically developed to address this gap [9]. The PEM-CY targets children and adolescents aged 5 to 17 years, while Young Children’s Participation and Environment Measure (YC-PEM) targets children under 5 years. Furthermore, the PEM-CY includes elements of the fPRC, a characteristic not observed in other instruments assessing participation.

Studies on Participation and Environment in children and adolescents with cerebral palsy (CP) consistently show that participation tends to be higher in home contexts and lower in school and community settings [2], especially among individuals with greater levels of motor impairment [10]. Evidence indicates that environmental barriers—such as physical inaccessibility, negative attitudes, and lack of institutional support—restrict participation regardless of motor function, whereas facilitating factors—such as family support, environmental adaptations, and assistive technologies—are associated with greater social engagement [11, 12].

Children and adolescents with CP have the same aspirations, desires, and perspectives regarding participation in social activities as their peers without disabilities. However, research shows that the opportunities available to them are often unequal, mainly due to barriers related to the physical, social, or attitudinal environment [2, 13]. Existing evidence on participation in children and adolescents with CP is dispersed across studies that use different conceptual frameworks and measurement instruments, often focusing exclusively on participation without simultaneously examining environmental factors. Furthermore, many instruments do not allow for a comprehensive comparison between different contexts (home, school, and community) or do not distinguish between frequency and involvement, which limits a deeper understanding of how participation occurs in daily life. As a result, evidence on participation patterns, environmental barriers and supports, and families' perspectives remains fragmented, limiting a comprehensive understanding of how these dimensions interact across home, school, and community settings. The PEM-CY is uniquely positioned to address these dimensions simultaneously; however, no literature review to date has synthesized evidence from studies using this instrument in children and adolescents with CP.

Addressing this gap requires not only methodological rigor but also alignment with the priorities and lived experiences of those directly affected. Patient and Public Involvement (PPI) brings patients, caregivers, and users of health services into the research process as partners, strengthening the relevance, transparency, and accessibility of research findings [14, 15]. Involving family members of children and adolescents with CP at key stages of the review process supports participatory science principles by valuing experiential knowledge and strengthening the applicability of evidence to real-world contexts. From this perspective, the objective of this scoping review is to synthesize evidence on participation and environmental factors in children and adolescents with CP, as assessed using the PEM-CY, and to identify factors influencing participation across home, school, and community settings, in partnership with a PPI group, to ensure that the review reflects family priorities and experiences.

## Methods

### Study design

This scoping review was structured according to the guidelines provided by the Joanna Briggs Institute (JBI) [16]. The review protocol was registered on the Open Science Framework (https://doi.org/10.17605/OSF.IO/VPQM5). The reporting of this review follows the *Preferred Reporting Items for Systematic Reviews and Meta-Analyses extension for Scoping Reviews (PRISMA-ScR)* guidelines [17]. Additionally, this study included a Public and Patient Involvement (PPI) group who were actively involved throughout the entire review process, including defining the research questions, refining the search terminology, validating the results, and co-interpreting the findings, in accordance with principles of meaningful and collaborative PPI engagement [14].

### Review stages

#### Stage 1 – Establishment of the Public and Patient Involvement (PPI) group and the co-production approach

This scoping review included a PPI group composed of seven members: three brazilian mothers/caregivers of children and adolescents with CP, three physiotherapists, and one occupational therapist. Caregivers were purposely recruited from a previous study on cerebral palsy in which they had completed the PEM-CY, and were selected based on their experience caring for children with CP across different functional levels and age groups.

The PPI group, including caregivers, was actively involved across key stages of the scoping review. Their contributions were obtained through structured meetings conducted [online/in person], each lasting approximately 25 min. Discussions were guided by predefined topics relevant to each stage of the review, and feedback was documented through detailed notes taken by the research team. The collected input was organized and synthesized, and decisions were reached through consensus between researchers and PPI members.

Based on the structure of the PEM-CY, which encompasses dimensions of participation (frequency, involvement, and desire for change) and environmental contexts (home, school, and community), caregivers contributed to refining the interpretation of these components within the review. Specifically, they helped identify which dimensions of participation were most meaningful in each context, as well as to understand how different levels of participation may interact positively or negatively across home, school, and community, considering contextual and environmental factors.

#### Stage 2 – Contribution to the formulation of the research questions

The research questions were developed through collaborative discussions with the PPI group. The group contributed valuable perspectives regarding the environmental contexts in which they and their children are embedded. Accordingly, the following questions were formulated:

- What information is available in the literature regarding participation and environment in the home, school, and community settings among children and adolescents with CP assessed by the PEM-CY?
- What personal, caregiver, and environmental factors are associated with participation among children and adolescents with CP across home, school, and community settings, as assessed by the PEM-CY?

#### Stage 3 – Support in identifying search terms and strategies for locating relevant studies

Although the academic team conducted the database search, the selection of keywords and search terms was informed by a consultation meeting with the PPI group. The mothers reviewed preliminary keyword lists and provided suggestions regarding the language commonly used by caregivers and families. This helped align the terminology used in the search strategy with real-world expressions and ensured that relevant concepts were not overlooked due to overly technical formulations. Their contribution enhanced the clarity, inclusiveness, and sensitivity of the search strategy.

Systematic searches for eligible studies were conducted in the following databases in May 2025: Medline/PubMed, Embase, Scopus, Web of Science, Lilacs, and CINAHL. In all databases, combinations, and variations of keywords were used—terms that were selected in collaboration with the PPI group as best representing the review topic. A manual search was also carried out using the reference lists of the included articles. A sample search strategy is provided in the Appendix.

Original studies that used the *Participation and Environment Measure for Children and Youth (PEM-CY)* in children and adolescents with CP were included. Only studies published from 2011 onward—the year of the first publication on the PEM-CY—were considered. The PEM-CY is a parent/caregiver-reported questionnaire designed for children and adolescents aged 5 to 17 years, which assesses participation and environmental factors across home, school, and community settings over the previous four months. Comprising 25 participation items and 45 environmental items, the instrument is distinguished by grouping participation into categories of activities (e.g., socializing using technology), rather than focusing on isolated tasks. For each item, frequency is scored from 0 (never) to 7 (once or more per day), and involvement from 1 (minimally involved) to 5 (very involved). Desire for change is assessed dichotomously (yes or no), allowing identification of whether the need for change relates to frequency, involvement, or variety of activities, with final scores ranging from 0 to 100% [9].

The environmental component includes the analysis of supports and barriers, expressed as percentages of items rated as “usually helps” or “usually yes” (supports) and “usually makes harder” or “usually no” (barriers), reflecting factors that facilitate or hinder participation. In addition, it encompasses composite measures such as environmental helpfulness, based on the responses “usually makes harder,” “sometimes helps/sometimes makes harder,” and “usually helps” or “not an issue”; environmental resources, derived from the responses “usually no,” “sometimes yes/sometimes no,” and “usually yes” or “not needed”; and overall environmental support, which integrates these components. All these indicators are calculated by summing the scores obtained for each item in relation to the maximum possible score and are subsequently converted into percentages (0–100%).

#### Stage 4 – Engagement in discussions regarding study selection

Although the initial screening (titles, abstracts, and full texts) was conducted by the academic reviewers, the PPI group contributed by discussing which types of studies were most relevant for families. Since the home, school, and community settings were predefined by the structure of the PEM-CY, PPI contributors did not influence the selection of settings themselves. Instead, they contributed to interpreting the relevance of participation experiences and environmental factors across these contexts. Although PPI members did not participate directly in the screening process, their input informed discussions among reviewers in cases of uncertainty, particularly when studies partially met inclusion criteria but addressed relevant aspects of participation or environmental context. This contributed to more consistent and contextually grounded inclusion decisions and reinforced the practical applicability of the selected studies.

The inclusion criteria for this review were based on the *Population, Concept, Context* (PCC) framework recommended by the JBI, which aims to guide the search strategy for scoping reviews. Table 1 below presents the PCC elements for this review, which focused on children and adolescents aged 5 to 17 with CP.

**Table 1:** Population, concept, and context

**Population**	Children and adolescents aged 5 to 17 years with cerebral palsy at all levels of the Gross Motor Function Classification System (GMFCS), and their parents or caregivers
Concept	Participation and environment across the three settings (home, school, and community) in children and adolescents with cerebral palsy, as assessed by the PEM-CY
Context	Original articles, regardless of study design. No language restrictions

### Inclusion criteria

This review included analytical and descriptive observational studies, quasi-experimental, and experimental studies involving children and adolescents (5–17 years old) with CP (GMFCS levels I–V, who constituted between 50 and 100% of the study sample). Eligible studies were those published in any language and that used the PEM-CY instrument to address the topics of participation and environment.

### Exclusion criteria

Excluded from this review were: systematic reviews (although reference lists of relevant articles were examined to ensure all eligible studies were identified), general discussion papers about participation measures that did not present new scientific evidence, and qualitative studies, as the focus of this review was on quantitative outcome measures. Conference abstracts, research letters, editorials, opinion pieces, letters to the editor, notes or project reports, and study protocols were also excluded.

### Study selection

Three independent reviewers (JMMS, VAP, and JBF) performed the initial screening and selection of studies. Any disagreements were resolved through discussion with senior researchers (EL and KA) to reach a consensus regarding the search strategy.

#### Stage 5 – Description of the preparation process for data mapping and development of the data extraction framework

Two independent authors (JMMS and JBF) screened titles and abstracts. Disagreements were resolved by consulting a third senior reviewer (EL) to achieve consensus.

### Study screening

Titles and abstracts were independently screened by the same two reviewers (JMMS and JBF). Studies that did not meet the inclusion criteria were excluded, and reasons for exclusion were documented in a flowchart, prepared according to the PRISMA guidelines [17]. Any discrepancies at any stage of the selection process were resolved through discussion with three senior reviewers (EL, KA, and HRL).

### Study characteristics

Key characteristics extracted by the reviewers (JMMS and JBF) included: authors, year of publication, study objective, country (upper-middle-income countries—UMIC; and/or high-income countries—HIC) [18], language, study type, sample characteristics (age range or mean age, sex, and GMFCS level), and methods (study design, data collection instruments, and statistical analyses used).

Since the home, school, and community participation domains were predefined by the use of the PEM-CY, the PPI group, particularly the mothers, contributed by reviewing the contextual relevance and interpretation of the extracted findings. They emphasized the importance of understanding environmental supports and barriers in a way that reflected caregivers’ real experiences. Their contribution supported a more family-centered interpretation of the extracted findings.

#### Stage 6 – Mapping, interpretation, and validation of the results

In this stage, two reviewers (JMMS and JF) independently extracted the data, and the results were subsequently discussed in a video conference meeting with the PPI group. Two additional senior researchers (EL and KA) were consulted in cases of uncertainty during the critical assessment of results related to Participation and Environment across the three settings (home, school, and community) and regarding the data extraction process.

### Mapping of participation and environment data

Participation data were extracted from the studies according to PEM-CY scoring domains. For “frequency” and “involvement”, mean values were extracted. The “desire for change” domain was operationalized as a binary response (yes/no) for each activity, where parents indicated whether they wished for a change in frequency, involvement, or variety of participation; the mean percentage of activities for which change was desired was then reported. Regarding factors that may influence participation, we extracted information only for variables that showed statistically significant associations, indicating whether a factor may enhance or restrict participation within a given setting. Environmental data were extracted as percentage values reported in the included studies, indicating the proportion of environmental factors identified as either barriers or supports. Five aspects were considered: “barriers”, “supports”, “environmental aids”, “available resources”, and “general environmental support”.

### Interpretation and validation of the results

The PPI group played a key role in the interpretation and validation of the mapped data. During virtual and in-person meetings, the caregivers reviewed emerging patterns and were invited to comment on whether the findings aligned with their lived experiences. The mothers shared perspectives on how environmental factors influenced participation experiences in daily life across different settings, including challenges related to accessibility, transportation, and social support. They also emphasized that frequent participation in activities did not always correspond to meaningful involvement. These discussions enriched the interpretation of the findings by providing additional contextualization and supporting a more family-centered understanding of the evidence. This collaborative stage exemplified co-production, allowing the families’ perspectives to directly shape the understanding of the evidence.

#### Stage 7 – Co-development of the discussion and implications

The final stage involved structured discussions with the PPI group to refine the review’s implications and conclusions. The mothers contributed reflections on how the findings could inform clinical practice, family-centered interventions, and future research directions. In particular, they emphasized the need for conclusions to clearly reflect differences in participation across home, school, and community settings, and to highlight the role of environmental barriers and supports in shaping participation. They also suggested that the implications should be presented in a way that is meaningful and understandable for families.

**Fig. 1:**
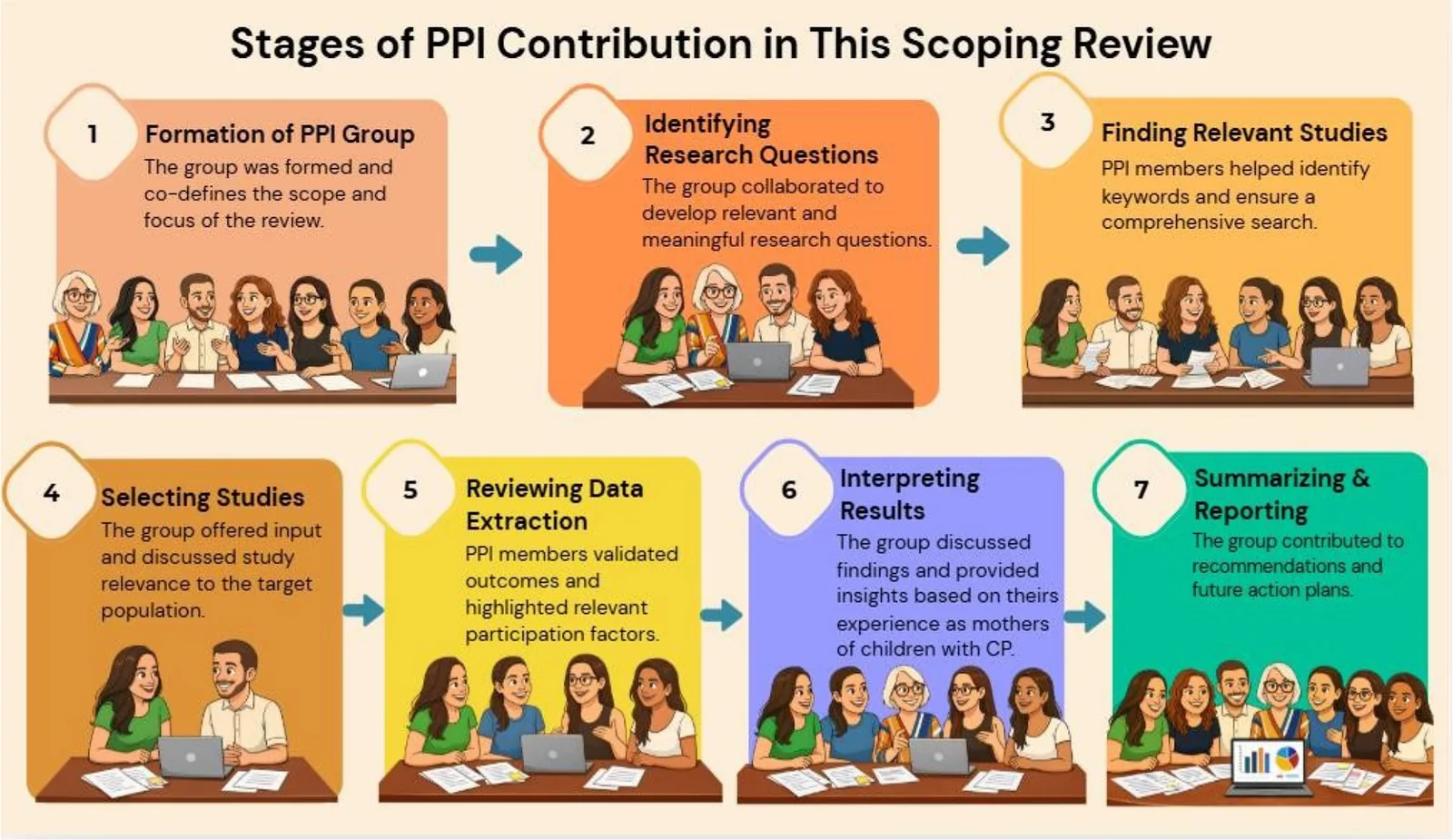
Stages of PPI contribution in the review

This input contributed to refining the wording and focus of the conclusions, ensuring that they were grounded in both the synthesized evidence and the perspectives of caregivers, while also enhancing their clarity and relevance to family contexts. By aligning the findings with practical considerations, this process supported the development of conclusions that are meaningful for caregivers, clinicians, and researchers. Figure 1 illustrates the stages of the review along with the respective participants. Figure 1 illustrates the stages of the review along with the respective participants.

## Results

**Fig. 2:**
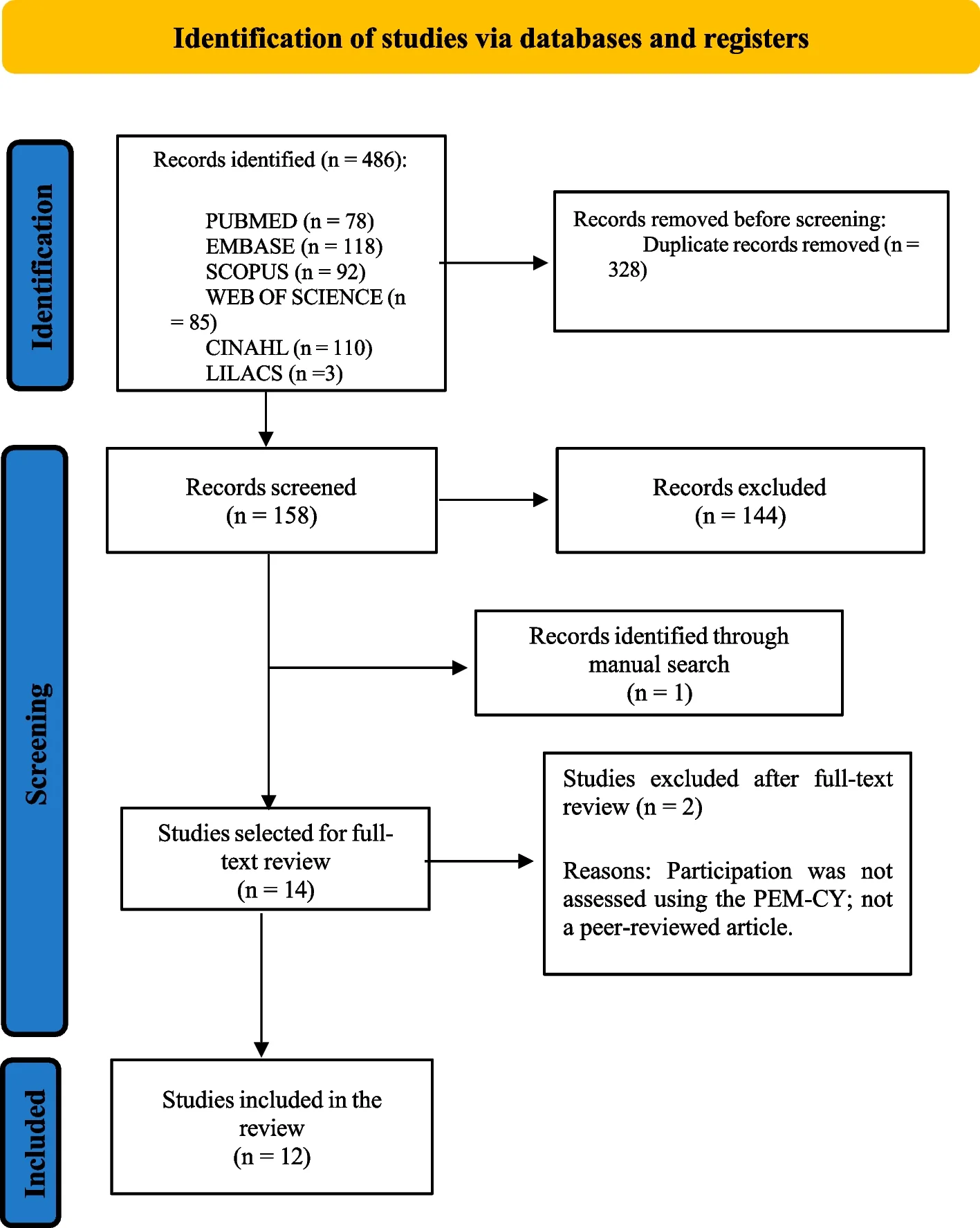
Flowchart based on the studies included in the review, 2025

The search returned 486 records, of which 328 were duplicates, leaving 158. After independent screening of titles and abstracts, 144 studies were excluded, and consensus was reached to select 13 articles. During full-text review of these 13 articles, one additional study was identified through manual reference checking, resulting in 14 manuscripts included for full-text reading. Subsequently, 2 studies were excluded for not meeting the eligibility criteria, leaving a total of 12 studies for this scoping review. Figure 2 presents the study inclusion flowchart and reasons for exclusion, based on PRISMA guidelines.

A total of 894 children and adolescents participated in the studies included in this review, with sample sizes ranging from 12 to 244 participants. Of these, 634 (70,9%) had a diagnosis of cerebral palsy (CP). The mean age of participants with CP was 9.9 years, and males were more prevalent (*n* = 345; 54,4%), most classified as GMFCS Level I (*n* = 171; 26,9%). Among the studies that included children with CP, two also involved participants with other conditions, although CP accounted for more than 50% of the sample in both studies [19, 20]. A summary of the 12 reviewed studies is presented in Table 2. Six studies were conducted in low- and middle-income countries, including Brazil [20–22] and Serbia [2, 23, 24], while the other six were developed in high-income countries (HICs).

**Table 2:** Characteristics of studies on the PEM-CY

Author (year)	Country (income) and language	Study objective	Sample characteristics	Methods
Mitchell, Ziviani, Boyd (2014) [27]	Australia (HIC), English	To quantify the extent to which physical, personal, and environmental characteristics are associated with physical activity in a group of independently ambulant children with unilateral CP	102 children with CP. Mean age: 11 y and 3 m. 52 male and 50 female, GMFCS levels I (*n* = 44) and II (*n* = 58)	Cross-sectional, descriptive, and analytical study. The following characteristics were measured: physical activity, mobility, functional strength, age, sex, and environment. Multiple linear regression analysis was performed
Milićević, Nedović (2018) [2]	Serbia (UMIC), English	To analyze and compare participation at home and in the community between children with cerebral palsy and children with typical development	244 children: 110 with CP and 134 with typical development. Children with CP: 61 male and 49 female, GMFCS levels I (*n* = 15), II (*n* = 29), III (*n* = 22), IV (*n* = 29), and V (*n* = 15)	Cross-sectional, descriptive, analytical, and comparative study. Covariance (ANCOVA) was analyzed to compare differences in Participation and Environment scores
Milićević (2020)	Serbia (UMIC), English	To investigate home participation among children with and without cerebral palsy, aged 7 to 18 years, and the effect of the environment on this participation	244 children: 110 with CP and 134 with typical development. Children with CP: 61 male and 49 female, GMFCS levels I (*n* = 15), II (*n* = 29), III (*n* = 22), IV (*n* = 29), and V (*n* = 15)	Cross-sectional, descriptive, analytical, and comparative study. Chi-square test was used for categorical variables, and the Mann–Whitney test was used to compare groups
Milićević, Nedović (2020)	Serbia (UMIC), English	To examine the relationship between family-centeredness in service delivery and home and community participation of children with cerebral palsy	244 children: 110 with CP and 134 with typical development. Children with CP: 61 male and 49 female, GMFCS levels I (*n* = 15), II (*n* = 29), III (*n* = 22), IV (*n* = 29), and V (*n* = 15)	Cross-sectional study in which Spearman correlation was performed between parents’ satisfaction with their children's current participation and their perceptions of the care provided by rehabilitation services
Takaki, Nitta, Kusumoto (2021) [19]	Japan (HIC), English	To identify factors that limit community participation in children with disabilities	100 children with physical disabilities, 51 CP. Mean age: 11.9 (± 3.6) years. 48 male and 52 female. GMFCS levels were not specified	Cross-sectional study. Spearman correlation was performed between PEM-CY domains and child characteristics. Mann–Whitney test was used to compare groups with low and high gross motor function, as well as between those with and without use of medical care. Multiple regression analysis was also conducted
Feitosa, et al. (2021) [21]	Brazil (UMIC), English	To investigate the feasibility and preliminary effects of an intensive, individualized, goal-directed training program for adolescents with cerebral palsy	12 adolescents with CP. Mean age: 15 y and 2 m. 5 male and 7 female, GMFCS levels I (*n* = 5), II (*n* = 1), III (*n* = 2), and IV (*n* = 4)	Intervention study based on functional goal-setting with 12 adolescents with CP. The PEM-CY was used as a secondary outcome measure to assess home participation. All outcome measures were assessed at four time points: one month before the intervention, two days before, two days after, and one month following the intervention. The Friedman test was used to compare results before and after the intervention
Barbosa, Silva e Portela Júnior (2022) [20]	Brazil (UMIC), Portuguese	To characterize participation and mobility in children and adolescents with cerebral palsy living in the interior of the Amazon region	30 children with CP. Mean age: 7.06 (± 4.06). 20 male and 10 female, GMFCS levels I (*n* = 1), II (*n* = 5), III (*n* = 7), IV (*n* = 8), and V (*n* = 9)	Cross-sectional and descriptive study. Total scores for Participation and Environment were obtained, and items related to participation at home and in the community were specifically analyzed
Aljuhani et al. (2025) [13]	Saudi Arabia (HIC), English	To assess the level of participation of children with cerebral palsy in home and community settings and to investigate factors associated with this participation	100 children: 50 with CP and 50 with typical development. Children with CP: mean age of 10.1 (± 2.42). 28 male and 22 female, GMFCS levels I (*n* = 2), II (*n* = 19), III (*n* = 18), IV (*n* = 8), and V (*n* = 3)	Prospective, analytical, and comparative study. Differences between groups were assessed using chi-square and t-tests, and multivariate logistic regression analysis was performed
Di Leto et al. (2025) [28]	Italy (HIC), English	To investigate the quality of life (QoL) of Italian children with cerebral palsy, evaluating the predictive role of social participation, as well as motor and cognitive levels, on QoL dimensions	71 children with CP. Mean age: 8.9 (± 2.5). 31 male and 40 female, GMFCS levels I (*n* = 37), II (*n* = 15), III (*n* = 8), IV (*n* = 8), and V (*n* = 3)	Cross-sectional, analytical study. ANOVA or Kruskal–Wallis tests were used. Regression analyses were conducted to investigate whether baseline factors and PEM-CY scores predicted QoL domains
Silva, Galvão, Mello (2025) [22]	Brazil (UMIC), Portuguese	To understand parents’ perceptions of participation and the environment for children and youth who use wheelchairs	43 children: 35 with CP and 8 with other diagnoses. Mean age: 8.1 (± 3.0). 28 male and 15 female	Cross-sectional and descriptive study. Data were collected on frequency and involvement in activities for each item in the home, school, and community settings, as well as environmental factors such as facilitators, barriers, environmental support, environmental resources, and general environmental support
Testani et al. (2024) [25]	Canada (HIC), English	To describe participation frequency patterns in young children with cerebral palsy at two time points (preschool and school age), and to investigate associations between child and family characteristics in the preschool period and community participation frequency at school age	155 children with CP. Mean age: 4.4 (± 1.1) at preschool and 6.7 (± 0.7) at school age. 87 male and 68 female, GMFCS levels I (*n* = 69), II (*n* = 29), III (*n* = 16), IV (*n* = 15), and V (*n* = 24)	Longitudinal, descriptive, analytical, and comparative study. Two multiple linear regression models were performed: one to explain child-related factors and the other to explain family-related characteristics in relation to participation frequency at home and in the community
Pierce e Fiss (2025) [26]	United States (HIC), English	To examine differences in participation between children and adolescents with and without cerebral palsy across settings and individual characteristics	37 children: 18 with CP and 19 with typical development. Children with CP: Age groups – < 6 years (*n* = 5), 6–11 years (*n* = 8), 12–17 years (*n* = 5). 13 male and 5 female, GMFCS levels I (*n* = 1), II (*n* = 5), III (*n* = 5), IV (*n* = 4), and V (*n* = 3)	Cross-sectional, descriptive, analytical, and comparative study. Mann–Whitney tests were used to compare children with and without CP, GMFCS level, and gender. Kruskal–Wallis tests were applied to compare participation frequency across age groups and family income levels

*UMIC* Upper-Middle-income countries, *HIC* High-income countries, *GMFCS* Gross motor function classification system

Most of the studies were cross-sectional observational studies, except one intervention study conducted in Brazil [21] and one prospective cohort study from Canada [25]. Among the cross-sectional studies, five compared children and adolescents with CP to those with typical development [2, 13, 23, 24, 26]. Additionally, five studies employed predictive models of participation. One investigated factors associated with physical activity, including participation across the three PEM-CY settings [27]; another explored limiting factors to participation in children with disabilities, with CP making up the majority of the sample [19]. Other studies examined various associated factors [13], the predictive role of participation in different quality of life domains [28], and child and caregiver characteristics that may influence community participation frequency [25]. These data are presented in Table 2 of this review.

All PEM-CY domains were investigated; however, only five studies (41,7%) provided information on both participation and environmental aspects [2, 13, 20, 23, 28]. Eleven studies (91,7%) reported data on participation frequency [2, 13, 19–23, 25–28], nine (75,0%) reported on the level of involvement [2, 13, 19–23, 26, 28], and seven (58,3%) addressed the desire for change [2, 13, 20, 22–24, 28]. In addition to the PEM-CY participation domains, two studies (16,7%) also reported on the total number of activities performed [19, 20].

Regarding the settings assessed by PEM-CY, eleven studies (91,7%) included information related to the home setting [2, 13, 20–28], five (41,7%) reported on school participation [20, 22, 26–28], and ten studies (83,3%). provided information regarding community participation [2, 13, 19, 20, 22, 24–28]. These data are presented in appendix 1 of this review.

### Participation across settings

Across the included studies, participation patterns differed consistently across settings, with higher frequency and involvement at home and lower levels in the community [2, 13, 19–28]. Overall, both frequency and involvement were higher in the home setting, intermediate in school, and lowest in the community, indicating a clear gradient of participation across contexts.

In terms of average participation frequency, the highest levels were observed in the home setting and the lowest in the community setting. At home, frequency ranged from 3.49 to 6.40 (once or few times in the last month to a few times each week), while in the community, it ranged from 1.57 to 4.20 (once in the last four months to a few times in the last month). Involvement was also higher at home, with values ranging from 3.40 to 4.30 (representing somewhat involved to 4), compared to 3.16 to 4.13 (representing somewhat involved to 4) in the community. The average percentage of desire for change was higher in the community setting compared to school and home. Regarding the environment domain, more resources and greater overall support were reported in the home environment compared to the community. These findings are also presented in appendix 1.

School participation appeared to occupy an intermediate position between home and community. Although fewer studies assessed this setting, available evidence indicates that both frequency and involvement at school are lower than at home but generally higher than in the community [20, 22, 26–28].

### Motor function

Gross motor function was associated with participation in four studies (*n*=250 participants with CP) [19, 23, 26, 28]. Three studies (*n*=179 participants with CP) reported that children and adolescents with lower levels of gross motor function exhibited lower participation across home [23, 26], school [26], and community [19, 26] settings. One study (n = 110 participants with CP) examined participation in the home setting and reported that children and adolescents with lower levels of gross motor function showed lower involvement in activities such as household tasks and technology-based interactions [23]. Additionally, one study (n = 71 participants with CP) found that lower levels of environmental support and helpfulness were perceived in the home setting than in school and community settings [28].

### Environmental factors and between-setting differences

Environmental supports and barriers to participation were reported in five studies (*n*=261 participants with CP) [2, 13, 20, 23, 28]. Three studies (*n*=211 participants with CP) reported descriptive data on environmental supports in the home [2, 20, 28], school [20, 28], and community [2, 20, 28]. Two studies (*n*=160 participants with CP) comparing children and adolescents with CP and typically developing peers identified physical demands [13, 23] cognitive demands [13, 23], social demands [23], and physical layout [23] as barriers within the home environment for children and adolescents with CP [13, 23]. In the community setting, one study (*n*=50 participants with CP) reported community safety as a barrier to participation [13]. The same study reported a lack of available programs and services and limited access to personal transportation as community barriers, and limited availability of equipment, materials, and financial resources as home barriers [13].

The home environment was described as more favorable and as having higher levels of support in three studies [2, 13, 20], lower levels of support in one study [28], and was the sole setting investigated in another study [23]. Two studies assessed levels of support in the school setting [20, 28]. One study reported higher levels of support at school than in the home environment [20], whereas the other found higher levels of support in the home environment than at school [28]. Both studies reported higher levels of support in the school setting than in the community setting [20, 28]. Four studies evaluated the community setting [2, 13, 20, 28]. In three of these studies, the community setting was reported to provide lower levels of support than the home setting [2, 13] and lower levels of support than both the home and school settings [20].

### Physical activity

One study (*n*=102 participants with CP) reported that higher levels of objectively measured physical activity, assessed using accelerometry, were associated with greater participation frequency across home, school, and community settings [27].

### Health complexity

In one study involving 51 children and adolescents with CP, the need for feeding tubes and respiratory care was associated with reduced participation, particularly in community-based activities [19].

**Fig. 3:**
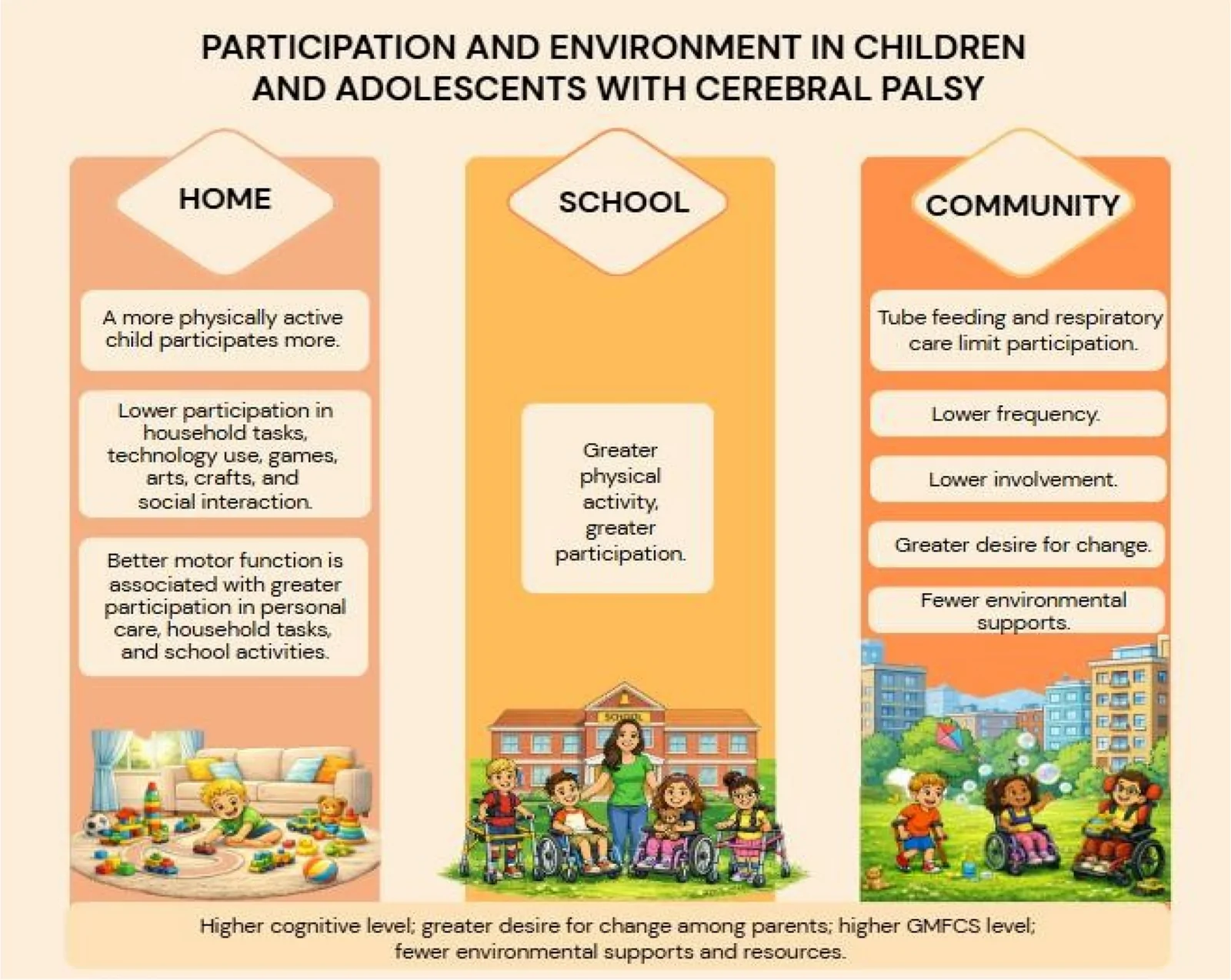
Summary of results from studies presenting predictive models of participation. Source: Prepared by the authors based on the included studies, 2025

Figure 3 summarizes the results from studies that presented predictive models of participation. Appendix 2 presents the descriptive and analytical results found in the studies included in this review.

## Discussion

This scoping review synthesized evidence on participation and environmental factors affecting children and adolescents with CP, as measured by the PEM-CY. This review was co-produced with a PPI group, whose involvement enhanced the practical and social relevance of the findings. Most included studies focused on home and community settings and mainly addressed participation frequency and involvement. Overall, few studies investigated factors associated with participation. Among these studies, the use of feeding tubes, the need for respiratory care, lower motor function, and lower levels of physical activity were associated with lower participation.

Regarding the contexts in which the included studies were conducted, six were conducted in upper-and middle-income countries and six in high-income countries. The predominance of studies conducted in middle- and high-income countries highlights a significant gap in the literature concerning the application of the PEM-CY in low-income settings. This concentration may be linked to greater availability of research funding, better infrastructure, and wider adoption of standardized instruments in such countries. Conversely, the absence of studies from low-income countries limits our understanding of how contextual, socioeconomic, and cultural variables influence the participation and environment of children and adolescents with CP, indicating the need for future investigations in more vulnerable settings.

In terms of sample characteristics, the 12 studies included in this review reported a predominance of male participants, a finding consistent with other systematic reviews [29, 30]. Regarding functional levels, GMFCS level I was most common, representing children with less functional limitations who can walk independently in most settings with few restrictions and without assistive devices [31]. This may positively influence participation outcomes, as children with greater mobility tend to be more involved in activities, especially outside the home [32, 33]. These findings suggest that the current literature has focused predominantly on children with higher levels of motor function, potentially neglecting the experiences of children with higher functional limitations (GMFCS levels IV and V). Furthermore, although some included studies enrolled participants across all GMFCS levels, few explored differences in participation according to functional severity. Future research should conduct subgroup analyses across GMFCS levels to better understand participation patterns among children and adolescents with different functional abilities.

Current research on the PEM-CY revealed a stronger focus on the frequency and involvement dimensions, with less attention paid to the desire for change and the environmental domain. This is a relevant gap, as the desire for change may reflect key areas of dissatisfaction for families and children regarding the quality of participation, and environmental factors are critical for identifying and addressing barriers and supports [4]. A more comprehensive understanding of participation therefore requires greater attention to both contextual influences and family perspectives, rather than focusing primarily on how often children participate or how involved they are in activities.

Opportunities for participation appear to be associated with the context in which activities occur, with domestic environments generally offering more favorable conditions than community settings. Frequency and involvement were generally higher at home than in the community, a pattern consistent with prior studies [34–37]. The home is typically more controlled, adapted, and supported by caregivers and family members, which may facilitate task modification and promote engagement in daily activities [2, 20, 23]. However, children with CP still participate less than their typically developing peers even within the home environment [2, 13, 23]. Reduced participation in technology-based socialization and digital games, for instance, may point to technological barriers, cognitive accessibility issues, or even parental stigma regarding the child’s capabilities. In contrast, lower participation in community settings was accompanied by fewer environmental supports and resources [2, 13, 20]. Previous research have identified physical, social, and attitudinal barriers—including inaccessible transportation and urban environments, limited support in public spaces, stigma, bullying, discrimination, and ineffective inclusion policies—as important obstacles to participation [11, 35, 38–40]. Together with challenges related to motor, cognitive, communication, and sensory impairments [1, 3, 13], these environmental barriers may restrict autonomy and participation and help explain the high levels of caregiver desire for change reported in community contexts [2].

Environmental factors appear to play a central role in shaping participation opportunities for children and adolescents with CP. Evidence from the included studies and previous literature suggests that the environment can either facilitate or restrict participation depending on resource availability, social attitudes, and physical accessibility [13, 28, 41, 42], highlighting the important role of environmental factors in shaping participation opportunities among children and adolescents with disabilities [41, 42]. Home environments were generally perceived as more favorable, whereas community participation proved to be particularly sensitive to environmental constraints. This pattern suggests that families may compensate for external barriers by adapting routines and expanding opportunities within the home environment, while broader structural limitations persist in community contexts. Therefore, the interaction between clinical limitations and environmental characteristics appears to significantly influence participation, highlighting the need for interventions that move beyond child-centered approaches and prioritize environmental adaptation, service accessibility, and inclusive community planning.

The school setting was also evaluated. Participation frequency was higher than in the community but lower than at home [20, 26–28]. However, involvement showed mixed results: one study indicated higher school involvement [20], while another found no significant differences between home, school, and community [28]. These findings suggest that while schools provide regular opportunities for participation, the quality and extent of engagement may be influenced by contextual factors within the educational environment. Previous studies have reported that barriers such as financial and transportation constraints, cultural beliefs regarding the educational potential of ambulatory students with CP [43], limited training of school staff [43, 44], and limited access to augmentative communication resources [44] may restrict participation in school activities. Discussions with the PPI group highlighted that parents may have less direct knowledge of their child's participation at school than in home or community settings, often relying on information provided by the child and/or teachers. This observation may be important when interpreting caregiver-reported school participation outcomes.

Although most studies were cross-sectional and used descriptive or comparative approaches, five studies that employed predictive models stand out, offering more robust insights into the factors associated with participation in this population. These studies consistently identified variables related to motor and cognitive abilities, environmental conditions, and overall health as key factors related to participation in daily activities. For instance, in a study investigating how physical, personal, and environmental characteristics relate to physical activity in children with CP, higher levels of physical activity were associated with greater participation across home, school, and community settings [27]. These findings align with existing literature highlighting physical activity as a catalyst for social inclusion and functional autonomy [4, 45]. However, given the predominantly cross-sectional nature of the included studies, these associations should be interpreted with caution, as they do not establish causal relationships. Longitudinal studies are needed to better understand the directionality of these relationships.

Moreover, clinical complexities such as the need for feeding tubes or respiratory care were found to restrict participation, particularly in community settings [19]. Higher complexity is typically associated with lower functioning in children with CP [46]. Such findings suggest that a child’s dependency on health care or equipment may limit spontaneous mobility and participation, emphasizing the need to consider clinical support as a central variable in inclusion policies.

The studies included in this review consistently identified environmental supports and barriers across home [2, 13, 28], school [28], and community settings [2, 13, 28], suggesting that contextual factors play a relevant role in shaping participation opportunities. These findings are consistent with previous literature indicating that environmental adaptations are as important as individual capabilities [11, 33]. Although the studies included in this review were not designed to determine the relative contribution of different factors, evidence from previous research suggests that environmental factors may have a particularly strong influence on participation among children and adolescents with disabilities [41, 47].

Additionally, one study identified participation itself as a predictor of perceived QoL [28], reinforcing the view that participation is central to well-being in children with disabilities [4]. In that study, higher cognitive levels were associated with a greater parental desire for change, suggesting that this desire may stem not only from the child’s limitations but also from heightened family expectations and future planning [42]. These findings emphasize the importance of family-centered approaches that consider both objective functional aspects and the subjective perceptions and goals of families. 

Although this review focused on quantitative studies using the PEM-CY, participation and environmental factors involve subjective experiences that may not be fully captured by quantitative measures alone. Constructs such as families’ “desire for change” reflect underlying perceptions and contextual challenges that require qualitative exploration. This is particularly relevant given the PPI group’s emphasis on lived experiences and family priorities. Future research integrating qualitative approaches may further enhance the understanding of participation in children and adolescents with CP. 

Despite the contributions of this scoping review, some limitations of the study itself should be considered. The adopted design was not intended to assess the methodological quality of the included studies or to establish causal relationships, which limits inferential interpretations. In addition, the relatively small number of included studies may have restricted the breadth of the synthesis and the identification of more consistent patterns in the literature. 

The interpretation of these findings should also consider the characteristics of the included samples. Although studies covered the full GMFCS spectrum, participants with lower levels of motor impairment were predominant, which may have influenced the observed patterns of participation. As a result, the experiences of children with more severe motor limitations may be underrepresented. In addition, limited evidence from low-resource settings may affect the applicability of the findings across different socioeconomic contexts, where environmental barriers and access to services can vary substantially. 

Overall, the findings of this review highlight important gaps in the current PEM-CY literature. Although participation frequency and involvement were consistently investigated across studies, environmental factors and families’ desire for change received comparatively less attention. This imbalance is noteworthy, as participation is shaped not only by children's functional characteristics but also by the contexts in which daily activities occur. Furthermore, evidence from studies using predictive models indicates that participation is associated with multiple interacting factors. Future research should therefore expand the investigation of environmental influences and family priorities, incorporate qualitative approaches, examine participation across different GMFCS levels, and include underrepresented low-resource settings to strengthen the evidence base and improve the applicability of findings across diverse contexts. 

## Conclusion

The findings of this review suggest that children with CP exhibit lower levels of participation frequency and involvement than their typically developing peers. Environmental supports and barriers were identified across all evaluated settings, highlighting the potential role of contextual factors in promoting or restricting opportunities for engagement. Participation frequency was generally lower in community settings. 

Additionally, the findings indicate that while differences in participation are evident, caregivers' perceptions of activity adequacy and desire for change tend to be relatively similar between children with CP and their typically developing peers, which may reflect both an adaptation of family expectations and an acceptance of the contextual limitations they face. These results underscore the importance of interventions that address not only the child’s individual capabilities but also family dynamics, environmental characteristics at home, and broader community challenges. Strategies that strengthen supports and reduce barriers in both home and community contexts are essential for expanding participation opportunities, fostering inclusion, development, and improved quality of life. Lastly, future research should further explore the interactions between contextual and functional factors and assess interventions focused on environmental modification and family empowerment as pathways to enhance participation. 

## Supplementary Information

Supplementary Material 1.

## Data Availability

All data generated or analysed during this study are included in this published article [and its supplementary information files.

## Abbreviations

CP: Cerebral palsy
PEM-CY: Participation and Environment Measure for Children and Youth PPI: Patient and Public Involvement
JBI: Joanna Briggs Institute
PCC: Population, Concept, Context
GMFCS: Gross Motor Function Classification System UMIC: Upper-middle-income countries
HIC: High-income countries
