# Supplementary material for: What shapes participation in cerebral palsy? A Co-produced PEM-CY scoping review with mothers with lived experience
Source: BMC Pediatr. 2026 Jul 27;26:1055. doi: 10.1186/s12887-026-07282-8 (PMC13693240; doi:10.1186/s12887-026-07282-8)
Supplement: Supplementary file 1 [file 12887_2026_7282_MOESM1_ESM.docx]

Appendix 1. Descriptive and analytical results from the studies included in this review.

| **Author (year)** | **Domain** | **Seccion** | **Descriptive results** | | | | **Analytical results** |
| --- | --- | --- | --- | --- | --- | --- | --- |
| Mitchell, Ziviani, Boyd (2014) | Frequency | Home  School  Community | Frequency  Home: 5.20 (±0.60)  School: 3.30 (±1.00)  Community: 2.3 (±0.90) | | | | Greater participation at home, school, and in the community was associated with higher levels of moderate to vigorous physical activity (p < 0.001). This association was strongest in the community setting. Lower levels of participation across all three environments were associated with higher levels of physical inactivity (p < 0.001). |
| Milićević, Nedović (2018) | Frequency  Involvement  Desire for change  Supports | Home  Community | Frequency  Home: 6.14 (±0.65)  Community: 4.20 (±1.07)  Involvement  Home: 3.42 (±1.05)  Community: 3.16 (±1.07) | Desire for change  Home: 63.91 (±24.35)  Community: 72.55 (±23.12)  Supports  Home: 78.64 (±12.34)  Community: 71.57 (±12.38) | | | A statistically significant difference was found for all Participation and Environment scores at home and in the community (p < 0.001). Children with CP participated in fewer activities in both settings. Parents of children with CP expressed a greater desire for changes in their children's participation in both settings (p < 0.001). |
| Milićević (2020) | Frequency  Involvement  Desire for change | Home | By home item | | | | Significant differences were found in participation frequency for household chores, socialization through technology, and playing computer or video games (p < 0.001). Children with CP showed higher frequency in indoor play and games, but the difference was not statistically significant. Except for the item "watching TV, videos, and DVDs," engagement scores were lower among children with CP (p < 0.001). Strong correlations were found between gross motor function and managing self-care, household tasks, and school-related activities. Significant differences were found in physical, cognitive, and physical layout demands (p < 0.001). Children in both groups tended to engage more in passive, sedentary, and less demanding activities |
| Milićević, Nedović (2020) | Desire for change | Home  Community | Desire for change  Home: 63.91 (±24.35)  Community: 72.55 (±23.12) | | | | Higher levels of parental satisfaction with their child's participation at home were correlated with higher perceptions of family-centeredness, specifically in the domains of general and specific information provision. |
| Takaki, Nitta, Kusumoto (2021) | Number of activities  Frequency  Involvement | Community | Number of items: 4.00 (±2.10)  Frequency: 2.80 (±1.10)  Involvement: 3.60 (±1.20) | | | | Significant positive correlations were found between child height and weight and the number of activities and level of engagement. Significant negative correlations were observed between child and parent age and participation frequency. Significant differences were found in the number of activities and participation frequency between groups with higher vs. lower levels of gross motor function, and between those with and without use of medical care. In multiple regression analysis, the number of activities was influenced by the use of medical care. Tube feeding and respiratory care were found to limit community participation. |
| Feitosa, et al. (2021) | Frequency  Involvement | Home | Frequency: 6.30 – 6.40  Involvement: 3.90 – 4.30 | | | | No significant differences were observed in frequency (p = 0.929) and engagement (p = 0.129) in home participation. |
| Barbosa, Silva e Portela Júnior (2022) | Frequency  Involvement  Desire for change  Number of activities  General environmental support | Home  School  Community | Frequency  Home: 3.49 (±1.57)  School: 2.80 (± 0.86)  Community: 1.57 (±0.82)  Involvement  Home:4.16 (±0.93)  School: 4.53 (±0.74)  Community: 4.13 (±1.25)  Desire for change  Home: 32.4 (±25.80)  School: 27.2 (±33,80)  Community: 34 (±31.20) | | Number of activities  Home: 6.13 (±2.42)  School: 2.60 (±0.63)  Community: 4.07 (±1.51)  General environmental support  Home: 45 (±9.00)  School: 41 (±8.00)  Community: 37 (±8.00) | | No inferential tests were conducted. |
| Aljuhani *et al*. (2025) | Frequency  Involvement  Desire for change  Environment (supports, barriers, resources) | Home  Community | No descriptive results available | | | | Community  Frequency: Significant differences were found in: neighborhood outings, organized physical activities, classes and courses, religious or spiritual gatherings, and being with other children in the community.  Involvement: Significant differences in: organized physical activities, classes and courses, and religious or spiritual gatherings.  Desire for Change:  Significant differences in: religious or spiritual gatherings and being with other children in the community.  Environment:  Barrier: Community safety  Resources: A lack of available programs and services, as well as limited access to personal transportation.  . |
|  |  |  |  |  |  |  | Home  Frequency: Significant differences in: computer and video games, arts, crafts, music, and hobbies, and being with other people.  Involvement: Significant differences are observed in: computer and video games, arts, crafts, music, and hobbies; social interactions with other people; and household chores.  Desire for Change:  Significant differences in arts, crafts, music, and hobbies, as well as personal care.  Environment: Barriers: Physical, cognitive, and social demands Resources: Limited availability of equipment, materials, and financial resources |
| Di Leto *et al*. (2025) | Frequency  Involvement  Desire for change  Environment (general support, resources, supports) | Home  School  Community | No descriptive results available | | |  | Frequency:  Significantly higher frequency of participation at home compared to school, and at school compared to the community.  Involvement:  No significant differences observed.  Desire for Change:  Significantly greater desire for change at home than at school.  Environment: Lower levels of environmental support and helpfulness were perceived at home compared to other settings.  Predictive Factors  -Higher cognitive levels were associated with greater parental desire for change.  -Higher GMFCS levels were associated with fewer environmental resources and supports. |
| Silva, Galvão, Mello (2025) | Frequency  Involvement  Desire for change | Home  School  Community | By Item Across the Three Settings  Home: The highest frequencies were found for being with other people (6.90±0.20) and watching TV (1.90±3.00); the lowest frequency was observed for household chores (2.00±2.90). The lowest engagement was observed during homework (4.00±1.30). The greatest desire for change was related to school preparation (72.10%) and household chores (58.10%).  Community: Participation frequency was low across all activities. The highest frequency was for neighborhood outings (3.90±2.60), and the lowest for attending classes or courses (0.10±0.80). The highest engagement was observed in unstructured physical activities (4.80 ± 0.60). The greatest desire for change was in organized physical activities (79.10%). | | School: Highest frequencies were found for classroom activities (5.70±2.50) and being with peers outside the classroom (5.20±3.00). Highest engagement was in field trips and school events (5.00±0.00). The greatest desire for change was related to school outings (62.80%). | | No inferential tests were conducted. |
| Testani et al. (2024) | Frequency | Home  Community | Frequency:  Preschool period:  Home = 5.20  Community = 2.80  School period:  Home = 5.40  Community = 2.80 | |  | | Attitudinal characteristics such as “strengths in prosocial behavior” and “some strengths in prosocial behavior” were associated with increased community participation compared to those classified as having “difficulties in prosocial behavior.” |
| Pierce e Fiss (2025) | Frequency  Involvement | Home  School  Community | Frequency:  Home = 4.82  School = 3.24  Community = 2.39 | | Involvement:  Home = 3.80  School = 3.72  Community = 3.47 | | Significantly greater frequency of participation at home and in the community was observed in typically developing children. Significantly greater participation frequency at home and school was seen in children classified as GMFCS levels I, II, and III. Older children showed significantly higher frequency of participation at school. |
